# Development of a lecithotrophic pilidium larva illustrates convergent evolution of trochophore-like morphology

**DOI:** 10.1186/s12983-017-0189-x

**Published:** 2017-02-08

**Authors:** Marie K. Hunt, Svetlana A. Maslakova

**Affiliations:** Oregon Institute of Marine Biology, University of Oregon, P. O. Box 5389, Charleston, OR 97420 USA

**Keywords:** Pilidium, Trochophore, Convergence, Nemertea, Larva, Marine invertebrate, Development, Imaginal discs, Lecithotrophy

## Abstract

**Background:**

The pilidium larva is an idiosyncrasy defining one clade of marine invertebrates, the Pilidiophora (Nemertea, Spiralia). Uniquely, in pilidial development, the juvenile worm forms from a series of isolated rudiments called imaginal discs, then erupts through and devours the larval body during catastrophic metamorphosis. A typical pilidium is planktotrophic and looks like a hat with earflaps, but pilidial diversity is much broader and includes several types of non-feeding pilidia. One of the most intriguing recently discovered types is the lecithotrophic *pilidium nielseni* of an undescribed species, *Micrura* sp. “dark” (Lineidae, Heteronemertea, Pilidiophora). The egg-shaped *pilidium nielseni* bears two transverse circumferential ciliary bands evoking the prototroch and telotroch of the trochophore larva found in some other spiralian phyla (e.g. annelids), but undergoes catastrophic metamorphosis similar to that of other pilidia. While it is clear that the resemblance to the trochophore is convergent, it is not clear how *pilidium nielseni* acquired this striking morphological similarity.

**Results:**

Here, using light and confocal microscopy, we describe the development of *pilidium nielseni* from fertilization to metamorphosis, and demonstrate that fundamental aspects of pilidial development are conserved. The juvenile forms via three pairs of imaginal discs and two unpaired rudiments inside a distinct larval epidermis, which is devoured by the juvenile during rapid metamorphosis. *Pilidium nielseni* even develops transient, reduced lobes and lappets in early stages, re-creating the hat-like appearance of a typical pilidium. Notably, its two transverse ciliary bands can be ontogenetically linked to the primary ciliary band spanning the larval lobes and lappets of the typical planktotrophic pilidium.

**Conclusions:**

Our data shows that the development of *pilidium nielseni* differs remarkably from that of the trochophore, even though their larval morphology is superficially similar. *Pilidium nielseni*’s morphological and developmental features are best explained by transition from planktotrophy to lecithotrophy in the context of pilidial development, rather than by retention of or reversal to what is often assumed to be the spiralian ancestral larval type — the trochophore. Development of *pilidium nielseni* is a compelling example of convergent evolution of a trochophore-like body plan within Spiralia.

**Electronic supplementary material:**

The online version of this article (doi:10.1186/s12983-017-0189-x) contains supplementary material, which is available to authorized users.

## Background

Nemerteans (ribbon worms) are a phylum of ~ 1300 described species [1] of primarily marine spiralians (lophotrochozoans) characterized by an eversible proboscis within a rhynchocoel. Like most benthic marine invertebrates, nemerteans have a biphasic life history with benthic adults and planktonic larvae. Their larvae are usually classified as either planuliform larvae (“direct developers”) or pilidia (“indirect developers”), but these two categories encompass a diverse array of developmental modes.

Planuliform larvae are named for their superficial resemblance to cnidarian planulae (uniform ciliation, specifically), and are found in the Hoplonemertea and the Palaeonemertea (e.g. [2, 3]), two of the three major lineages. Their development is comparatively “direct,” with the larva gradually becoming more worm-like as it transitions into its adult form, although the two groups display significant differences in development, and certain characteristics of indirect development are found in hoplonemerteans [4–6]. The third major lineage, the Pilidiophora [7, 8], which comprises the sister taxa Heteronemertea and Hubrechtiidae, is named for its idiosyncratic pilidium larva, a long-lived planktotroph which typically resembles a deer-stalker cap with the earflaps pulled down (from Greek *pilos (πῖλος),* or *pilidion (πιλίδιον) —* a type of brimless conical cap). Pilidial development is “maximally-indirect” [9]; the juvenile is formed by a series of discrete paired invaginations of the larval epidermis, called imaginal discs, as well as unpaired juvenile rudiments possibly derived from the mesenchyme. A total of eight juvenile rudiments, including three pairs of imaginal discs and two unpaired rudiments, gradually fuse together around the larval gut to form the complete juvenile, which ultimately emerges from—while it simultaneously ingests—the larval body in a dramatic catastrophic metamorphosis [10 and references therein].

The basic elements of pilidial development are conserved in all pilidia; each one develops via a sequence of imaginal discs and rudiments and undergoes catastrophic metamorphosis. However, the shape of the pilidium, the orientation of the juvenile anteroposterior (AP) axis relative to the larval AP axis, and the reported number and sequence of rudiments vary [2, 5, 10–18]. That said, the reported variation in the number of juvenile rudiments formed during the development of different species may be partially attributed to ambiguity in terminology; “imaginal discs” and “juvenile rudiments” are often used interchangeably in the literature (e.g. [16, 19]) as, historically, it was believed that the juvenile developed via seven imaginal discs, all of which invaginated from the larval epidermis (e.g. [20, 21] and references therein). Note here, that we use “imaginal discs” only to describe the paired discs formed by epidermal invaginations, while “juvenile rudiments” will include imaginal discs, as well as the unpaired rudiments not formed by invaginations [10]. These terms indicate tissue origin and formation, so it is important to distinguish between them.

Beyond alterations in morphology, it is also increasingly clear that pilidiophorans have transitioned from a planktotrophic pilidium to a lecithotrophic pilidium repeatedly [2, 15, 22]. Since 2005, the number of pilidiophoran species known (or suspected) to have a non-feeding larva has increased from three (i.e. Desor’s larva, Schmidt’s larva and Iwata’s larva) to twenty [2, 15, 22–24]. Some of these are uniformly ciliated, while others, in addition to a complete covering of short cilia, have one or two circumferential ciliary bands of longer cilia which superficially resemble the prototroch and telotroch of trochophore larvae of other spiralians, e.g. annelids and molluscs [2, 15, 23, 24].

The subject of this study, a trochophore-like pilidium with an anterior “prototroch” and posterior “telotroch,” was dubbed *pilidium nielseni* [24] in honor of Claus Nielsen, for his provocative theories on the evolution of marine larval forms, in which the trochophore is considered the ancestral larva of spiralians [25–30]. *Pilidium nielseni*, which resembles a trochophore, is a lecithotrophic larva of an undescribed lineiform species (Lineidae, Heteronemertea, Pilidiophora) provisionally referred to as *Micrura* sp. “dark” [24]. Its mere existence prompts a central question in the trochophore debate — is the widespread occurrence of the trochophore morphology among spiralians due to the retention of an ancestral larval form, as Nielsen suggests, or did this larval body plan evolve multiple times independently [31–36]?

**Fig. 1:**
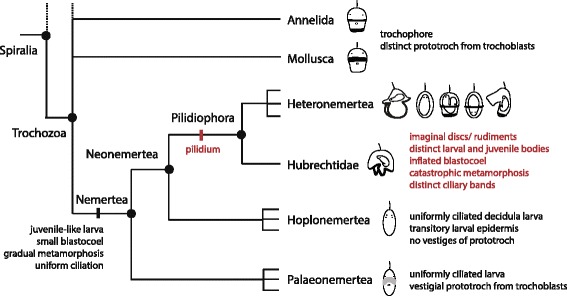
Evolution of larval development in nemerteans. The pilidium larva, defined here as a complex character including development via imaginal discs and juvenile rudiments, a larval body distinct from the juvenile body, an inflated blastocoel, catastrophic metamorphosis and distinct ciliary bands (*black*), is only found in the Pilidiophora. Hubrechtids possess a helmet-like planktotrophic pilidium with all of the listed features. Heteronemerteans display a great diversity of pilidia, including lecithotrophic forms, such as *pilidium nielseni*. Lecithotrophic pilidia lack inflated blastocoel; a few also lack distinct ciliary bands. Nevertheless, all pilidiophoran larvae possess other essential pilidial traits. Hoplonemerteans and palaeonemerteans possess uniformly ciliated juvenile-like larvae with small blastocoel and gradual metamorphosis. Hoplonemertean larvae possess a transitory larval epidermis (hence the name “decidula”), and lack any prototroch vestiges. At least one palaeonemertean genus, *Carinoma,* possesses a vestigial prototroch (*grey*), derived from the spiralian trochoblast lineage (including 1q^2^ cells). Parsimony suggests that pilidium evolved at the base of the Pilidiophora, while juvenile-like uniformly ciliated larvae are ancestral to Nemertea. Thus the distinct ciliary bands of pilidiophoran larvae (including those of *pilidium nielseni*) must have evolved independently from the ciliary bands (including the prototroch), of other spiralian larvae, even if the prototroch, and the trochophore larva defined by it, are shown to be ancestral to the Trochozoa, or Spiralia, as a whole. It is not possible to represent the immense diversity of spiralian larvae in this figure. The goal here is merely to highlight that a trochophore larva defined by the presence of a preoral differentially ciliated prototroch derived from the trochoblast lineage is found in at least some annelids and mollusks. Note that the eye spots, as depicted here, do not reflect morphology of any particular species, but simply mark the position of the juvenile head

Convincing evidence for a nemertean trochophore was conspicuously absent until 2004, when a vestigial prototroch was discovered in the palaeonemertean *Carinoma tremaphoros* [37, 38]. This discovery offered support for the view that a trochophore-like larva may have been ancestral to nemerteans. However, all palaeonemertean larvae (including *Carinoma*’s), and all hoplonemertean larvae are uniformly ciliated, and lack distinct ciliary bands (Fig. 1). Distinct ciliary bands are only present in the Pilidiophoran lineage. In light of the recent transcriptomic molecular phylogeny of the phylum [8]: (Palaeonemertea (Hoplonemertea; Pilidiophora)), it is most parsimonious to assume that a uniformly ciliated larva was ancestral to the Nemertea, and thus the ciliary bands of pilidiophoran larvae (planktotrophic pilidia and lecithotrophic larvae, such as *pilidium nielseni*, alike) evolved secondarily, and are unlikely to be homologous to the trochophore’s prototroch. This view is further supported by the differences in cell lineage [39], cell fate [SA Maslakova and G von Dassow: The trochoblasts in the pilidium larva break an ancient spiralian constraint to enable continuous larval growth and maximally-indirect development, in preparation.], and morphology and function of pilidial ciliary bands [40] compared to those of a trochophore. Furthermore, *pilidium nielseni,* despite its similarity to the trochophore larva, clearly belongs to a pilidiophoran species, and it undergoes catastrophic metamorphosis, like all other pilidiophorans. Although there is quite a bit of variation in larval morphology and configuration of ciliary bands within the Heteronemertea, currently there is no evidence to suggest that *pilidium nielseni* represents the ancestral condition for the Heteronemertea, the Pilidiophora, or the Nemertea as a whole. Within the Heteronemertea, typical hat-like planktotrophic pilidia are widely distributed, and it is the only known larval type in the Hubrechtidae, which suggests planktotrophy was likely ancestral to the Pilidiophora. From the functional point of view, one can explain imaginal discs and catastrophic metamorphosis of *pilidium nielseni* and other lecithotrophs, as a vestige of planktotrophic pilidial development. The alternative possibility (lecithotrophy ancestral to Pilidiophora) defies a functional explanation, and requires multiple independent origins of planktotrophic pilidia (once in the Hubrechtidae, and several times within the Heteronemertea), which seems unlikely given the high degree of complexity of form and function. To sum it up, the trochophore-like appearance of *pilidium nielseni* appears to be a case of striking convergence. However, the development of *pilidium nielseni* remains undescribed. To determine how much of the typical pilidial developmental pattern is conserved, and to understand the developmental basis of such evolutionary convergence, we described and illustrated the development of *pilidium nielseni* with light and confocal microscopy. We demonstrate that *pilidium nielseni* has a clear pilidial development with modifications related to lecithotrophy, and that the development of its ciliary bands differs from that of the trochophore. Our data illustrates an example of convergent larval morphology based on different developmental pathways.

## Results

**Fig. 2:**
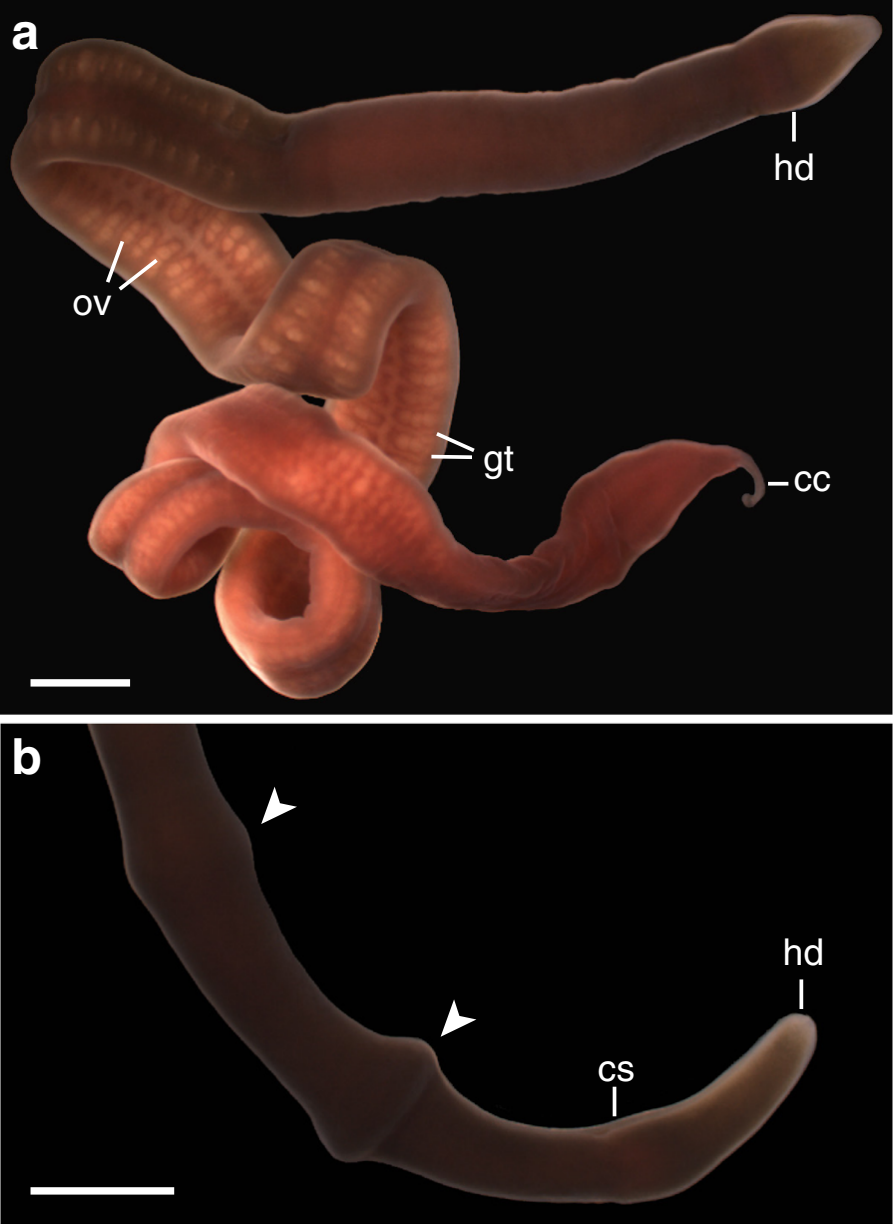
Adult morphology of *Micrura* sp. “dark.” **a**. Live adult (female) showing the caudal cirrus (cc) at the posterior end. Large oocytes inside the ovaries (ov) alternating with gut diverticula (gt) are visible through the body wall of the midbody region. **b**. Head end (hd) showing distinctive peristaltic waves (arrowheads) and lateral cephalic slits (cs). Scale bars 2 mm

**Fig. 3:**
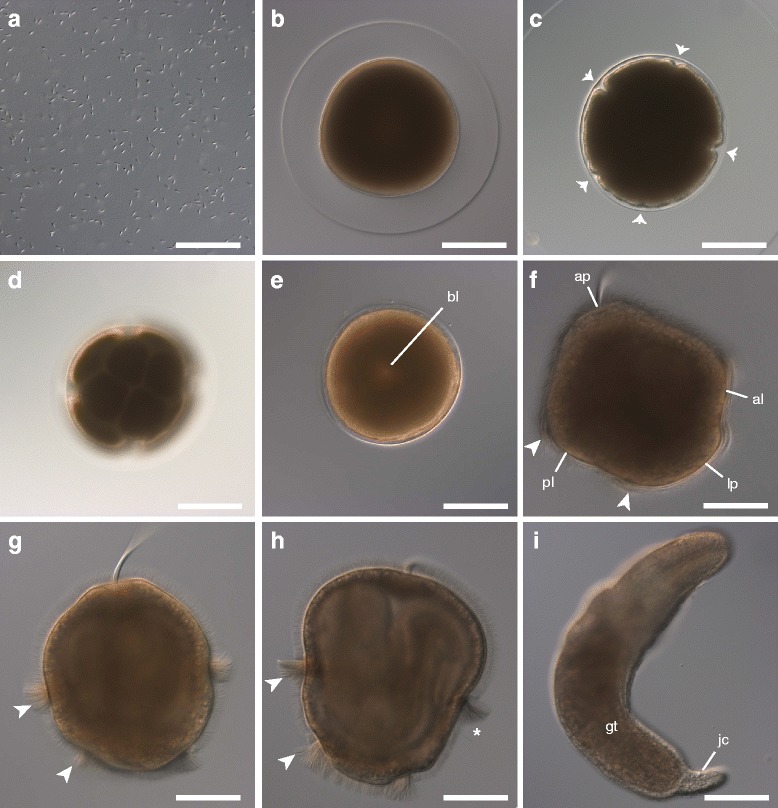
Lecithotrophic development of *Micrura* sp. “dark.” DIC images. **a**. Sperm with a compact head. **b**. Unfertilized oocyte. **c**. Furrowing (*arrows*) prior to cleavage in a fertilized oocyte. **d**. Eight-cell stage showing spiral arrangement of blastomeres. **e**. Ciliated gastrula with narrow blastopore (bl). **f**. Lateral view of “pileus” stage; note apical tuft (ap), transient lateral lappets (lp), anterior (al) and posterior lobes (pl), each fringed with longer cilia (*arrowheads*). **g**. Larva which has lost its lobes and lappets, taking on the characteristic *pilidium nielseni* shape with two circumferential ciliary bands (*arrowheads*). The cilia are fanned out during a brief arrest of ciliary beat, as is typical in *pilidium nielseni*’s stop-start swimming pattern. **h**. Lateral view, juvenile anterior to the left, showing the larval cirrus (*asterisk*) between the two transverse ciliary bands (*arrowheads*). The juvenile body is visible through the larval epidermis. **i**. A recently metamorphosed juvenile with its larval body in its gut (gt). Juveniles have a long cirrus (jc) at the posterior end. Scale bars 100 μm

We raised eleven cultures of *Micrura* sp. “dark” through metamorphosis, and seven more through the early developmental stages. Reproductive females are readily identified due to the relatively large size of the oocytes, which are visible through the body wall (Fig. 2a). Whether spawned freely through the gonopores or dissected out, oocytes are ~250 μm in diameter, opaque and pale orange in color, and have a distinct chorion (~265 μm in diameter) and jelly coat (~430 μm) (*n* = 6) (Fig. 3b). Reproductive males have noticeably pale gonads. The sperm (Fig. 3a) have a compact head ~5 μm long (*n* = 6), as is typical of species with external fertilization [41]. The rate of development is highly dependent on temperature, with cultures at 8 °C requiring at least 18 days to metamorphose, and cultures at 16 °C metamorphosing as early as 9 days after fertilization (Table 1). For simplicity, we will focus on describing the order and earliest appearance of significant developments of larvae raised in cultures kept at ~16 °C.

**Table 1:** Developmental timeline of *Micrura* sp. “dark”

Stage	Description	Earliest appearance (16 °C)	Earliest appearance (8 °C)
Furrowing	Embryo furrows at five to seven sites (Fig. 3c)	2.5 h	3 h
1st Cleavage	Embryo cleaves equally	3 h	4 h
Blastula	Blastula is slightly flattened along the animal-vegetal axis	15.5 h	22 h
Gastrula	Gastrula is somewhat flattened along animal-vegetal axis, becomes ciliated, and develops an apical tuft and a vegetal invagination (blastopore). Gastrulae may swim freely in advanced stages (Fig. 3e)	20.5 h	24 h
Cephalic discs	Paired cephalic discs invaginate (Fig. 4)	30 h	—
Cephalic and trunk discs	Paired trunk discs invaginate (Fig. 4)	42 h	—
Pileus stage	Larva develops transient lobes and lappets, the gut curves backward, the paired cerebral organ discs invaginate from the gut, and the proboscis and dorsal rudiment appear (Figs. 3f and 5). Ciliary bands appear as four segments which span each transient lobe and lappet (Fig. 6)	3 days	3 days
Torus stage	The head and trunk rudiments fuse around the base of the gut (Fig. 8). Ciliary band segments are re-arranged to form two complete transverse ciliary bands	4 days	—
Hood stage	Epidermis of trunk rudiment extends over the proboscis, but has not yet fused with the epidermis of the head rudiment, leaving a dorsal gap (Fig. 9)	6 days	—
Metamorphosis	The head and trunk rudiments fused to form a complete juvenile (Fig. 10). Juvenile erupts from and devours larval body in catastrophic metamorphosis	9 days	18 days

Dash represents missing data

**Fig. 4:**
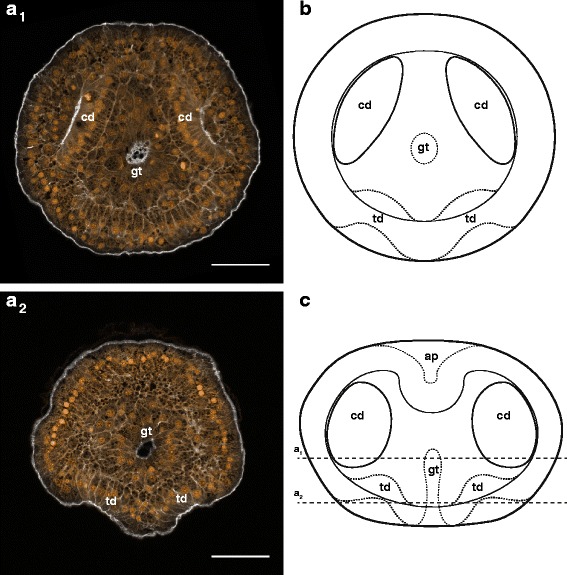
Invagination of cephalic and trunk discs in larvae of *Micrura* sp. “dark.” a_1_-a_2_ are confocal projections of a specimen stained with phalloidin (*white*) and propidium iodide (*orange*) and sectioned transversely (from apical to vegetal); anterior lobe is up. **a**_1_. A 1.95 μm slab showing the cephalic discs (cd) and the gut (gt). **a**_2_. Same individual as on a_1_, a 1.95 μm slab showing the trunk discs (td) invaginating from the larval epidermis. **b**. A diagram (apical view) summarizing a_1_-a_2_ (apical organ omitted for clarity). **c**. A diagram of the same stage as on a-b, showing a frontal view (apical up). Horizontal lines show approximate levels of the sections in a_1_-a_2_. Scale bars 50 μm


We did not observe the germinal vesicle in dissected or naturally spawned oocytes (likely due to the yolkiness of the eggs), or the formation of polar bodies, but in previously studied nemertean species, primary oocytes are released, undergo germinal vesicle breakdown upon contact with sea water, and produce polar bodies after fertilization (e.g. [10]). It is unclear how this occurs in *Micrura* sp. “dark.” After fertilization, eggs undergo equal spiral cleavage, with a distinct size difference between the animal and vegetal quartets at the eight-cell stage (Fig. 3d). However, without the polar bodies to mark the animal pole, it is unclear whether the micromeres are larger than the macromeres, as they are in other nemerteans with described development (e.g. [37] and references therein), or the other way around, as in most other spiralians. In most, but not all cultures, prior to first cleavage, we observed fertilized eggs exhibit five to seven transient furrows which gave the eggs the appearance of an orange in cross-section. This phenomenon was subtle and ephemeral; the furrows did not seem to fully separate the segments. In cases where we did not observe these furrows, we cannot be certain whether the culture passed through this stage particularly quickly, or skipped it entirely, but whether we did or did not observe furrowing did not seem to correlate with the success of the culture. The furrows lasted less than an hour, and faded within about an hour prior to first cleavage (Fig. 3c; Table 1). First cleavage occurs as soon as three hours after fertilization, but most often occurs after four hours (Table 1). This relatively large time range may be attributable to the length of time oocytes were exposed to sea water before insemination. Second cleavage occurs as soon as 30 min later, with most cleaving 4.5–5.5 h post-fertilization, and the 8-cell stage is reached as early as one and a half hours following that, though most cultures required two hours to progress from second to third cleavage. Subsequent cleavage stages are reached every hour, approximately, and a blastula forms within the first day, as early as 15.5 h after fertilization (Table 1). Embryos gastrulate and develop cilia several hours later, as early as 20.5 h after fertilization, resulting in an embryo which is somewhat flattened along the animal-vegetal axis, and features a small blastopore (Fig. 3e, Table 1). Larvae hatch from the chorion and begin swimming the next day, and the gut gradually elongates from the vegetal blastopore toward the apical tuft at the animal pole. The first two pairs of imaginal discs are apparent before the second day (Fig. 4, Table 1, Additional file 1 — Movie 1). The paired cephalic discs appear first, as early as 30 h after fertilization, and several hours later, are followed by the paired trunk discs. Both pairs of discs are formed by invaginations of the larval epidermis, and separate themselves from the larval body wall in an arc curving towards the sagittal plane of the larva. Soon afterwards, the gut beings to curve backwards over the trunk discs.

Additional file 1: Movie 1: Invagination of cephalic and trunk discs in larvae of *Micrura* sp. “dark.” A running z-projection movie of the confocal z-series used to make Fig. 4. Specimen stained with phalloidin (*white*) and propidium iodide (*orange*). Transverse sections (apical to vegetal), anterior lobe is up. Scale bar 50 µm. (MOV 11208 kb)


**Fig. 5:**
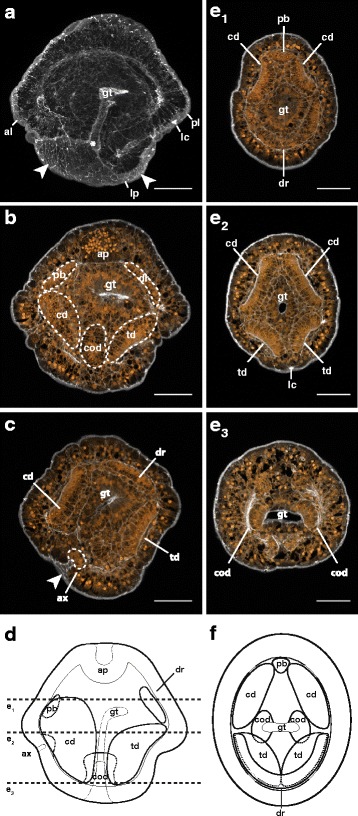
Anatomy of the “pileus” stage of *Micrura* sp. “dark.” a-c, e_1_-e_3_ are confocal projections of specimens stained with phalloidin (a) or phalloidin (*white*) and propidium iodide (*orange*). a-c sagittal sections, apical plate up, anterior lobe (al) to the left. e_1_-e_3_. Transverse sections of the same individual (from apical to vegetal), anterior lobe is up. **a**. A 29.9 μm-thick slab showing the opening of the blastopore (*asterisk*) between the lateral lappets (lp), the anterior (al) and posterior (pl) lobes, the lumen of the gut (gt), and the ciliated band (*arrowheads*) spanning the lateral lappet. Larval ciliary cirrus (lc) visible underneath the posterior lobe. **b**. Same individual as in A, a 1.95 μm slab showing the apical plate (ap), the gut, and the juvenile rudiments (*dashed outlines*) inside: one of the cephalic discs (cd), proboscis rudiment (pb), dorsal rudiment (dr), one of the trunk discs (td), and one of the cerebral organ discs (cod). **c**. A 1.95 μm slab showing the axil (ax, *dashed outline*), the ciliated band terminating in the axil (*arrowhead*), cephalic disc, dorsal rudiment, trunk disc and the gut. **d**. A diagram (lateral view) summarizing A-C (outer outline of the gut omitted for clarity). Horizontal lines show approximate levels of the sections on e_1_-e3. **e**_1_. A 1.95 μm slab showing the proboscis rudiment, paired cephalic discs, gut and dorsal rudiment. **e**_2_. A 1.95 μm slab showing paired cephalic discs and trunk discs, and the gut. **e**_3_. A 1.95 μm slab showing paired cerebral organ discs invaginating from the gut. F. A diagram (apical view) summarizing e_1_-e_3_ (outer outline of the gut omitted for clarity). Scale bars 50 μm

**Fig. 6:**
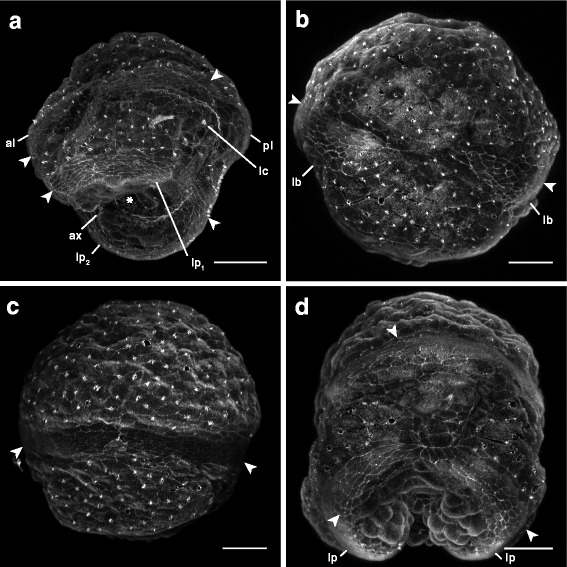
Development of the ciliary bands in the *pilidium nielseni* of *Micrura* sp. “dark.” Confocal z-projections of 3–5 day old larvae stained with phalloidin, and oriented with the apical plate up. Ciliary bands are marked by arrowheads. **a**. A slightly oblique lateral-vegetal view showing the “pileus” stage (3-day old). Anterior lobe (al) left and posterior lobe (pl) right. Posterior lobe can be identified by the position of the larval cirrus (lc). Shows the two separate segments of the future “telotroch” spanning the two lateral lappets (lp_1_, lp_2_) and the two segments of the future “prototroch” (*arrowheads*) spanning the larval lobes (lb). Blastopore is marked with an asterisk. **b**. Lateral view of a larva at the “pileus” stage. Shows the lateral gap between the two segments of the future “prototroch.” **c**. Lateral view of a larva several hours past the “pileus” stage. Shows the two “prototroch” segments (*arrowheads*) making contact. **d**. Frontal view of a larva several hours following the “pileus” stage, showing the formation of the complete “telotroch” as the ciliary bands spanning the lappets (lp) make contact. Scale bars 50 μm

**Fig. 7:**
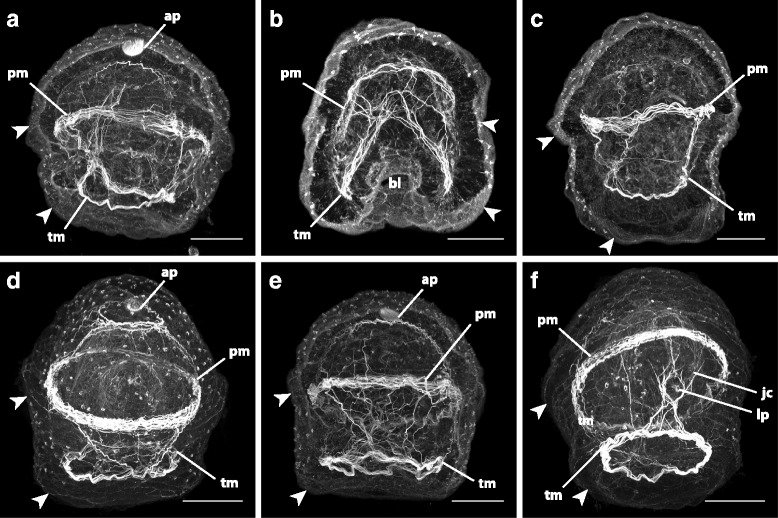
Larval muscles in *pilidium nielseni* of *Micrura* sp. “dark.” Confocal projections of specimens stained with phalloidin, apical plate (ap) up. **a**, **c**, and **e** are slightly oblique lateral views with the anterior lobe (and future juvenile anterior) to the left and the posterior lobe (future juvenile posterior) to the right. Ciliary bands are marked with arrowheads. **a**. A 92.95 μm stack showing “arcs” of muscles criss-crossing after they drop into the lappets from either side of the “prototroch” muscle (pm) band into the “telotroch” muscle (tm) band late in the “pileus” stage. **b**. A 26.0 μm slab showing a frontal (anterior) view of a late “pileus” stage, with the blastopore (bl) visible. **c**. A 48.1 μm slab of a specimen one day past the “pileus” stage showing the complete ring of circumferential muscles underlying the “telotroch,” formed in part by the “arcs” connected to the “prototroch.” **d**. A complete z-projection showing a frontal (anterior) view of a week-old specimen with a complete juvenile inside. Note the ring of muscles underlying the “telotroch” and the ring forming around the apical organ. Extensions of the apical organ muscles are descending towards the “prototroch.” **e**. A 106.6 μm stack (a frontal view) of a larva in the “torus” stage showing the increasing connections between the muscles of the apical ring, the “prototroch,” and the “telotroch.” **f**. Complete z-projection of a week-old larva with a fully-formed juvenile inside. A frontal (posterior) view showing the muscles around the larval pore (lp), just below the juvenile cirrus (jc). Scale bars 50 μm


The third day, larvae reach what we call the “pileus” stage, in reference to their hat-like shape which resembles a much reduced pilidium (Figs. 3f and 5, Additional file 2 — Movie 2); it has stubby lateral lappets and anterior and posterior lobes surrounding the vegetal blastopore. “Pileus”-stage larvae are ciliated over their entire surface, but also have a prominent apical tuft, and their reduced lobes and lappets are fringed with longer cilia. The longer cilia along the margins of the lobes and lappets are organized into four ciliary band segments, one spanning each larval lobe or lappet (Figs. 3f and 6). These ciliary band segments appear as several rows of small cells easily distinguishable from the larger cells of surrounding epidermis (Figs. 5a and 6). Early on, there is a distinct lateral gap on either side, between the ciliary bands of the anterior and posterior lobes (Fig. 6b), as well as anterior and posterior gaps separating the ciliary bands of the two lappets (Fig. 6a). The ciliary bands spanning each of the transient lappets extend from a tiny pair of epidermal invaginations between the anterior lobe and each lappet (Fig. 5a, c and d, Additional file 2 — Movie 2, Additional file 3 — Movie 3). These “pits” are in a position corresponding to the anterior axils (the growth zones) of the pilidium larva, which give rise to the ciliary bands in typical pilidia [19]. At this point, the cephalic discs are positioned above the transient anterior lobe, while the trunk discs are beneath the backward curve of the gut, along the posterior side of each lappet (Fig. 5b-d). At the same time, the unpaired dorsal rudiment forms along the inner pilidial epidermis dorsal/apical to the gut, and the unpaired proboscis rudiment forms between the cephalic discs (Fig. 5). The origin of these rudiments is uncertain, but perhaps, as is hypothesized for a typical pilidium, they are mesenchymal [10]. At any rate, they do not appear to form by invagination from the larval epidermis. Additionally, two shallow chambers outpocket from the basal portion of the gut (near the blastopore) between the developing cephalic and trunk discs on either side of the mid-sagittal plane. These invaginations of the gut elongate and form the cerebral organ discs (Fig. 5e_3_-f). The larva also begins to develop musculature at this point. First, it develops circumferential muscles underlying the ciliary bands of the transitory larval lobes (which later form the anterior ciliary band). As these muscles form, some begin to arc towards the posterior of the larva, extending into each of the transitory lappets, rather than encircling the larva (Fig. 7a-b). These extensions form an “arc” of muscle which follows the curve of the lappet (imagine two jump ropes, each wrapped around opposite sides of the larva, are allowed to droop into the lappet on its respective side).

Additional file 2: Movie 2: Sagittal sections showing anatomy of the “pileus” stage of *Micrura* sp. “dark.” A running z-projection movie of the confocal z-series used to make Fig. 5a-b. Specimen stained with phalloidin (*white*) and propidium iodide (*orange*). Scale bar 50 µm. (MOV 11239 kb)
Additional file 3: Movie 3: Transverse sections (from apical to vegetal) showing anatomy of the “pileus” stage of *Micrura* sp. “dark.” A running z-projection movie of the confocal z-series used to make Figure 5e_1_-e_3_. Specimen stained with phalloidin (*white*) and propidium iodide (*orange*). Scale bar 50 µm. (MOV 13778 kb)


Larvae begin to exhibit a distinctive start-stop swimming behavior between the third and fourth day of development; *pilidium nielseni* spiral forward led by the apical tuft, then abruptly stop and flare out the cilia, halting ciliary motion for a brief moment (Fig. 3g) before continuing on. At about the same time, the larval ciliary cirrus and an amniotic “larval pore” become apparent below what used to be the posterior larval lobe (now located between the two transverse ciliary bands) (Figs. 5a and e_2_). The larval pore is located just vegetal to the larval cirrus, and opens through the larval epidermis to the outside (Figs. 6a and 7f).

**Fig. 8:**
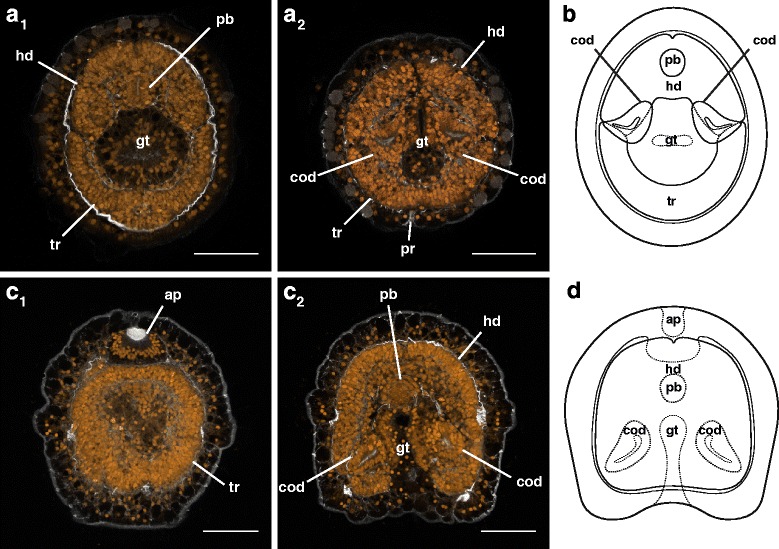
Anatomy of the torus stage of *Micrura* sp. “dark.” a_1_-a_2_ and c_1_-c_2_ are confocal projections of larvae stained with phalloidin (*white*), and propidium iodide (*orange*). a_1_-a_2_ are transverse sections (from apical to vegetal), juvenile anterior is up. c_1_-c_2_ are frontal sections (from posterior to anterior, apical is up). **a**_1_. A 1.95 μm slab showing the developing proboscis (pb) and fused pairs of cephalic and trunk discs, forming the head (hd) and trunk rudiments (tr), respectively. **a**_2_. The same individual as in a_1_. A 1.95 μm slab showing the head and trunk rudiments fused around the gut, forming the characteristic toroid of juvenile tissue. The cerebral organ discs (cod) are closed off from the gut. Note the larval pore (pr), which is associated with the larval cirrus (not visible on this slab). **b**. A diagram summarizing a_1_- a_2_. **c**_1_. (outline of the gut omitted for clarity). A 1.95 μm slab (frontal view) showing the apical organ and trunk rudiment. **c**_2_. A 1.95 μm slab (frontal view) showing the proboscis rudiment, the cerebral organ discs closed off from the gut. **d**. A diagram summarizing c_1_-c_2_ (outline of the gut omitted for clarity). Scale bars 50 μm


By the fourth day, typically, the lobes and lappets diminish and become indistinguishable, and corresponding halves of each ciliary band make contact with each other (Fig. 6c-d), with the ciliary band segments of the larval lobes forming a continuous anterior transverse ciliary band (the “prototroch”), and those of the lappets forming a continuous posterior transverse ciliary band (the “telotroch”) (Figs. 3g and 6d). As the ciliary bands reorganize, the “arcs” of muscle tracing the vanishing lappets begin to widen their curve, and the sides of the “arcs” extend towards each other (like the handles of each jump rope are being held further from each other, but closer to the handles of the opposite jump rope). The circumferential muscles underlying the “telotroch” weave through and around the widened curves, encircling the posterior end of the larva (Fig. 7c-d). Eventually, the sides of the “arcs” overlap each other, forming a cross of muscle at either end of the developing juvenile (Fig. 7a-d), and the widened curves of the “arcs” dropped from the “prototroch” muscles are more fully incorporated into developing circumferential muscles underlying the “telotroch” (Fig. 7c-d). Other muscle fibers extend between the “prototroch” and “telotroch,” further interconnecting the musculature. The dorsal rudiment becomes bi-layered, and spreads underneath the larval epidermis across the dorsal surface of the gut (Fig. 5b-c), and the cephalic discs envelop the proboscis rudiment as they fuse around it (Figure 5e_1_). The cephalic discs fuse together near the gut first, then continue to fuse anteriorly and around the proboscis into the fourth day (Fig. 8, Additional file 4 — Movie 4), forming the head rudiment. The trunk discs fuse with each other and the posterior end of the dorsal rudiment, forming the trunk rudiment. The dorsal rudiment also extends anteriorly over the gut towards the fusing cephalic discs. The cerebral organs close off from the gut, and are enveloped by the head and trunk rudiments as they fuse together around the opening of the gut, forming a toroidal juvenile rudiment (Fig. 8, Additional file 4 — Movie 4). This corresponds to the “torus” stage in the development of a planktotrophic pilidium [10].

Additional file 4: Movie 4: Anatomy of the torus stage of *Micrura* sp. “dark.” A running z-projection movie of the confocal z-series used to make Figure 8a_1_-a_2_. Specimen stained with phalloidin (*white*) and propidium iodide (*orange*). Transverse sections (apical to vegetal). Scale bar 50 µm. (MOV 13502 kb)


**Fig. 9:**
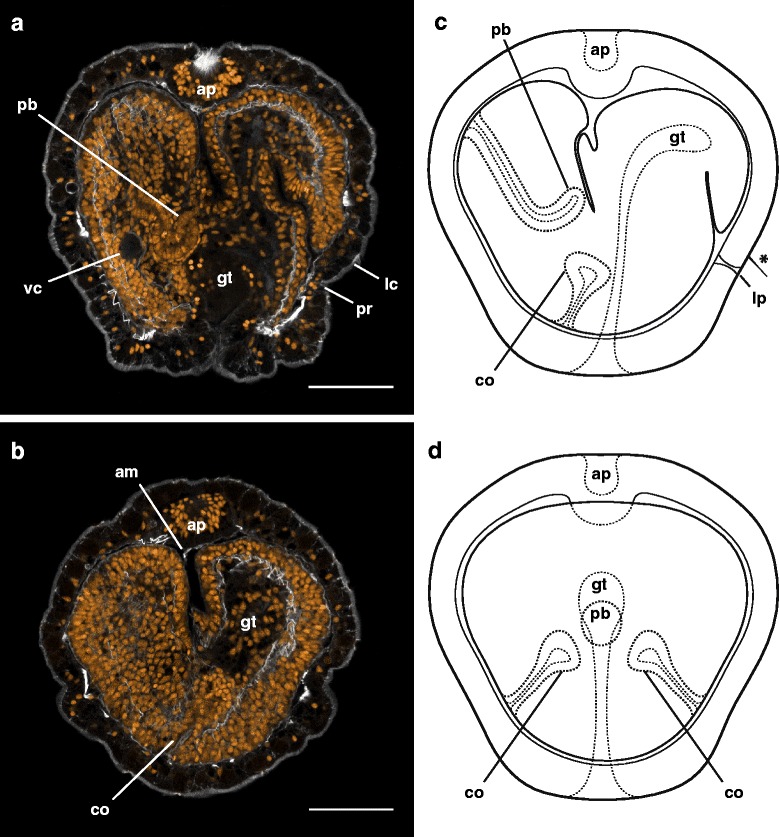
Anatomy of the hood stage of *Micrura* sp. “dark.” a-b are confocal projections of specimens stained with phalloidin (*white*) and propidium iodide (*orange*). Sagittal sections, apical plate (ap) up, juvenile anterior left. **a**. A 1.95 μm slab showing the proboscis (pb), the larval pore (pr) associated with the larval cirrus (lc), the ventral commissure (vc) and the lumen of the gut (gt). **b**. A 1.3 μm slab showing cerebral organ (co) opening laterally through the juvenile epidermis, the apical muscle (am) and the lumen of the gut. **c**. A diagram summarizing a-b (outline of the gut omitted for clarity). **d**. A diagram of the same stage from a frontal view. Scale bars 50 μm

By the fifth day, the cerebral organ discs penetrate the juvenile epidermis and open laterally (left and right) into amniotic space (Fig. 9), the fibrous cores of the cerebral ganglia are visible in the head rudiment, and the juvenile lateral nerve cords begin to extend from the head region into the trunk region, passing under the cerebral organ discs (Additional file 5 — Movie 5). Also, the circumferential muscle bands underlying the ciliary bands thicken, and the larvae begin to contract at their “trochs,” cinching in the larval body like tightening belts (Figs. 3h and 7d-e). Circumferential muscles also form around the apical organ (Fig. 7d-e). Some of these muscles originating around the apical organ begin to extend through the larva behind the cephalic discs, with fibers connecting to the “prototroch” and “telotroch” musculature (Fig. 9b, Additional file 5 — Movie 5). These fibers thicken and form a dense cord, the apical muscle, which allows the larva to pull in its apical tuft (Fig. 9b, Additional file 5 — Movie 5). Additionally, muscles which seem to anchor the cirrus of the juvenile to the larval body wall, just above the larval pore, begin to form.

**Fig. 10:**
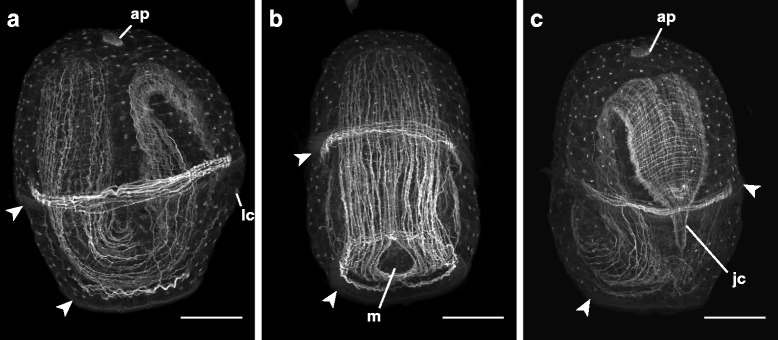
Complete juvenile within *pilidium nielseni* of *Micrura* sp. “dark.” Confocal z-projections of larvae stained with phalloidin, apical organ (ap) up. Larval ciliary bands, with associated circular muscle bands are denoted by arrowheads. **a**. Lateral view, with juvenile anterior to the left. **b**. Frontal (anterior) view showing juvenile anterior with juvenile longitudinal body wall muscles parted around opening of the larval/juvenile mouth (m). **c**. Frontal (posterior) view showing juvenile posterior end folded over, with the juvenile cirrus (jc). Scale bars 50 μm


At about six days, a ring of muscle forms around the larval pore just above the posterior muscle cross (Fig. 7f), which is the last major larval muscle group to form, and the juvenile begins to form longitudinal and circumferential muscles in the trunk region, which then expand anteriorly. The brain ring, which is made up of paired dorsal cerebral ganglia connected by the dorsal commissure and paired ventral cerebral ganglia connected by the ventral commissure (Fig. 9a), is apparent around the rhynchocoel, and the juvenile lateral nerve cords extend into the trunk region (Additional file 5 — Movie 5). About two days after the torus stage (as early as six days), larvae reach what corresponds to the “hood” stage in planktotrophic development [10], where the trunk rudiment, composed of the fused trunk discs and dorsal rudiment, extends over the proboscis rudiment, but is still separated from the head rudiment by a dorsal gap, which admits the apical muscle (Fig. 9, Additional file 5 — Movie 5). By eight days (and in a few instances, just under a week), the apical muscle extending through the larva degrades, and the head and trunk rudiments fuse dorsally, incorporating the proboscis and gut and forming a complete juvenile (Figs. 3h and 10). At this point, the larvae appear less opaque due to diminishing yolk reserves, so the complete juvenile is visible through the body wall (Fig. 3h). Approximately one day after the juvenile is completed, its epidermis becomes noticeably ciliated, while the circular and longitudinal muscles of the juvenile body wall extend from the more muscled posterior end into the head region.

Additional file 5: Movie 5: Anatomy of the hood stage of *Micrura* sp. “dark.” A running z-projection movie of the confocal z-series used to make Fig. 9a. Specimen stained with phalloidin (*white*) and propidium iodide (*orange*). Sagittal sections. Scale bar 50 µm. (MOV 11476 kb)


In as few as eight days, the juvenile begins to move within the larval body, pushing against it, and retracting from it. At the earliest, metamorphosis occurred in only nine days, and most individuals metamorphosed in fewer than 20 days. During its catastrophic metamorphosis, the juvenile extends against the larval body, distorting it, as its tail jabs between the ciliary bands near the lateral cirrus, as described by Maslakova and von Dassow [24]. Confocal imaging exposed a small larval pore open to the outside near the larval cirrus, which suggests that the juvenile may use this pore as an “escape hatch” during metamorphosis (Figures 6a_2_, 7f and 9a_1,_ 9b.

Newly metamorphosed juveniles have a length of ~500-600 μm in gliding, including a distinct caudal cirrus of ~50 μm (Fig. 2i). The cirrus is sometimes used as a sticky anchor while the juvenile extends its anterior end and writhes in the water. *Micrura* sp. “dark” juveniles have a pair of longitudinal cephalic slits, as is characteristic of adults of this species (and the entire family Lineidae), and a slight constriction separates the head from the rest of the body, which can appear somewhat bulbous while the stomach is engorged with the larval body (Fig. 3i).

## Discussion

**Fig. 11:**
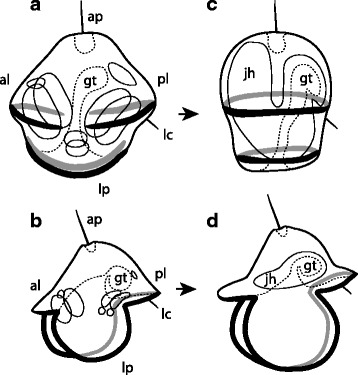
Development of ciliary bands and juvenile in pilidium nielseni. **a**. “Pileus” stage of *pilidium nielseni* with four separate segments of ciliary bands, corresponding to the anterior lobe (al), posterior lobe (pl), and the two lateral lappets (lp). Larval ciliary cirrus (lc) is underneath the posterior lobe. Dash line outlines apical organ (ap) and gut (gt). All eight juvenile rudiments (solid thin outlines) are already present. **b**. Comparable developmental stage of a typical planktotrophic pilidium. Continuous primary larval ciliary band spans the larval lobes and lappets, eight juvenile rudiments are present. Other ciliary bands of the typical planktotrophic pilidium (inner ciliary bands on lappets, esophageal ciliary ridges) are omitted for clarity. **c**. Advanced developmental stage of *pilidium nielseni* with a complete juvenile and two transverse circumferential ciliary bands. Juvenile head (jh) is at upper left. **d**. Comparable developmental stage of a typical planktotrophic pilidium. Juvenile head to the left. In both types of larvae juvenile posterior ends up near the larval ciliary cirrus

This is one of the first studies of non-feeding pilidiophoran development using modern microscopy methods [15–17]. The development of the lecithotrophic trochophore-like *pilidium nielseni* mirrors that of a typical planktotrophic pilidium. Initially, *pilidium nielseni* even takes the form of a highly reduced pilidium, developing transient lobes and lappets, a phenomenon which has not been observed in any other pilidiophoran species with non-feeding development. At this “pileus” stage, the ciliary bands span the lobes and lappets (though they do not form a continuous band), much like in a typical pilidium (Fig. 11a). The *pilidium nielseni* also shares its method of juvenile development; paired cephalic, trunk and cerebral organ discs, and unpaired proboscis and dorsal rudiments, arise and fuse together around the larval gut to form the juvenile (Fig. 11a, b). In a typical pilidium, the unpaired rudiments are possibly mesenchymal, or at least they do not obviously invaginate from the larval epidermis [10], and this also appears to be the case in *pilidium nielseni*. Finally, once the juvenile is complete, *pilidium nielseni* undergoes catastrophic metamorphosis, a quintessential pilidial trait. During metamorphosis, the juvenile backs out of the larva near (or possibly through) the larval amniotic pore, and draws the larval body into the shared mouth as it escapes [24]. Interestingly, the planktotrophic pilidium of *Maculaura alaskensis,* formerly known as *Micrura alaskensis* [42], has two amniotic pores underneath its posterior larval lobe —likely vestiges of the trunk disc invaginations [43] — and during metamorphosis, the *M. alaskensis* juvenile often emerges caudal end first in that vicinity, at the base of the posterior lobe [10]. In the planktotrophic, sock-like *pilidium recurvatum*, there is a single larval pore in a corresponding position (posterior to the mouth), and the juvenile has been observed to emerge near (possibly through) that pore, as well [44]. Another similarity between the *pilidium nielseni* and the typical planktotrophic pilidium is the larval ciliary cirrus — located, in *pilidium nielseni,* underneath the much reduced posterior lobe of the “pileus,” and associated with its larval pore (Fig. 11a, c). Correspondingly, a short larval cirrus is found underneath the posterior larval lobe in many typical planktotrophic pilidia ([10, 45], Maslakova, pers. obs., Fig. 11b, d]). However, as one might imagine, there are some deviations from typical pilidial development*.*

One of the most obvious differences between the typical pilidium and *pilidium nielseni* is lecithotrophy. In a typical hat-like planktotrophic pilidium, the ciliary bands generate currents while the lobes and lappets perform specialized movements to capture unicellular algae [38]. Likely, other kinds of planktotrophic pilidia, such as the mitten-shaped *pilidium auriculatum* and sock-shaped *pilidium recurvatum*, have developed feeding mechanisms suited to their individual morphologies [38]. The elaborate feeding structures and mechanisms required by planktotrophic pilidia are, of course, unnecessary for non-feeding pilidia, which begins to explain their simplified body plans.

All described free-swimming non-feeding pilidia are uniformly ciliated, or have one or two circumferential ciliary bands of long cilia in addition to short cilia covering the rest of the surface (e.g. [2, 12, 15, 22, 23]). They are also more streamlined, shaped like a prolate spheroid. Similar patterns of simplification and modification (with uniform ciliation or circumferential ciliary bands) are seen in the derived non-feeding larvae of some other taxa which ancestrally had a more complex planktotrophic larva, such as bryozoans (e.g. [46–49]), hemichordates (e.g. [50–52]), and echinoderms (e.g. [53–56]). It is thought that these patterns of ciliation and a streamlined body shape improve swimming ability, while complex larval feeding structures, such as ciliated bands extended on lobes and arms, increase hydrodynamic drag, thereby reducing swimming ability [53, 54, 57]. For non-feeding larvae, any pressure to feed efficiently is removed, and may be replaced by pressure to swim efficiently [53]. Accordingly, *pilidium nielseni*, much like the derived non-feeding larvae in other marine invertebrates, has reduced its feeding structures and reorganized its ciliary bands, converging on a trochophore-like body plan, and likely lessening the energy spent swimming.

Still, the trochophore-like appearance of *pilidium nielseni* is provocative, and there may be an impulse to draw a direct connection to the hypothetical ancestral trochophore larva of Spiralia (Lophotrochozoa or Trochozoa, depending on the interpretation). However, the “prototroch” and “telotroch” of *pilidium nielseni* are both positioned anterior to the blastopore (i.e. vestigial mouth), which retains its posterior/vegetal position. The prototroch of a true trochophore would also be anterior to the mouth, but the telotroch, if present, would surround the anus at the posterior end. Furthermore, the “prototroch” and “telotroch” of *pilidium nielseni* can be ontogenetically linked with the primary ciliary band of a planktotrophic pilidium, as they initially form along the lobes and lappets before wrapping around the larva as two circumferential ciliary bands (Fig. 11a, c). This is analogous to the re-organization of the ciliary bands during the auricularia-to-doliolaria transition in development of holothuroids [58, 59]. The ciliary band of typical pilidia functions very differently from the prototroch-metatroch pair in the opposed-band feeding mechanism described for some trochophores [38, 60–63] and is not homologous to the prototroch as a differentially ciliated band, so we can infer that the circumferential ciliary bands of *pilidium nielseni* are not homologous to the prototroch and telotroch in a typical spiralian trochophore, either (see Fig. 1). Additional substantiation may be provided by a cell lineage study of *pilidium nielseni*, which would clarify the relationship between its ciliary bands and those of a typical pilidium and trochophore, and determine which cell lineages contribute to the formation of the *pilidium nielseni* “trochs.”

Another distinction between planktotrophic pilidia and *pilidium nielseni* is the size of the eggs from which they arise. The eggs produced by *Micrura* sp. “dark” are ~250 μm in diameter, much larger than the 75–160 μm eggs of planktotrophic nemertean species [2] (Fig. 2b). The larger egg size is likely due to the proportionate abundance of yolk, which is later doled out into lipid granules dotting the larval epidermis [24]. The yolk provides enough nutrition for *pilidium nielseni* to develop a complete juvenile without ever needing to feed, which accounts for *pilidium nielseni*’s accelerated development to metamorphosis compared to that of a typical planktotrophic pilidium [10, 18, 45, 56, 64]. Relatively large eggs (150–350 μm) are also characteristic of other non-planktotrophic pilidia ([2] and references therein), and evolution of larger eggs is associated with lecithotrophy in other taxa as well [65]. Though these yolk-rich, relatively short-lived larvae are energetically more expensive to produce, the benefits of a shortened planktonic stage must outweigh the costs in these cases [66].

As described, the development of the juvenile within *pilidium nielseni* seems to align more closely to that of planktotrophic pilidia than of other non-feeding pilidia, but this may be due to the differences in interpretation by different authors, rather than biology. For instance, Iwata’s larva is described to develop via five imaginal discs (paired cephalic and trunk discs and a dorsal disc formed as epidermal invaginations), cerebral organ rudiments which invaginate from the stomodeum, and a proboscis which arises from the cephalic discs [12] (Table 2). A typical pilidium forms via three paired imaginal discs (formed as epidermal invaginations) and two unpaired juvenile rudiments (possibly mesenchymal) [10]. However, it was thought the proboscis was derived from the cephalic discs in typical pilidia, too [3, 20, 21], until recent data confirmed an early account by Bürger [67] of a separate proboscis rudiment [10]. Also, the stomodeal invaginations forming the cerebral organs in Iwata’s larva [12] are similar to the outpocketings of the gut near the blastopore in *pilidium nielseni*, and both are likely homologous to the lateral invaginations of the subumbrellar epidermis/esophagus which form the cerebral organ discs in typical pilidia [10]. All of these should be considered imaginal discs. Surprisingly, Iwata indicates that the dorsal disc invaginates above the paired cephalic discs [12], which is odd, as the dorsal disc appears above the trunk discs, then fuses with them to form the trunk rudiment in a typical pilidium [10]. It is possible that a review of *M. akkeshiensis*’s development with modern methods would yield different results, which are more aligned with development of other pilidia.

**Table 2:** Comparison of juvenile rudiment development in several lecithotrophic and one planktotrophic pilidium.

	Rudiments reported as imaginal discs	Other reported juvenile rudiments	Rudiments reported with uncertain origin	invaginating rudiments	Total # of juvenile rudiments	Source
*Micrura akkeshiensis*	5	3		7	8	
	Paired cd and td. Unpaired dd	Paired cor. Unpaired pb		Paired cd, td and cod. Unpaired dd		Iwata 1958 [12]
*Lineus ruber*	4	1	3	4	5–8	
	Paired cd and td	Unpaired pb	Paired cod. Unpaired pb	Paired cd and td		Martîn-Durán et al. 2015 [17]
	8			4	8	
	Paired cd, td, and cod. Unpaired dr and pb			Paired cd and td		Schmidt 1964 [68]
*Micrura rubramaculosa*	5			?	5	Schwartz and Norenburg 2005 [23]
*Micrura verrilli*	5			?	5	Schwartz 2009 [15]
*Micrura sp.* 803	6			?	6	Schwartz 2009 [15]
*Micrura sp.* “dark”	6	2		6	8	
	Paired cd, td and cod	Unpaired pb and dr		Paired cd, td and cod		This study
*Maculaura alaskensis* ^a^	6	2		6	8	
	Paired cd, td and cod	Unpaired pb and dr		Paired cd, td and cod		Maslakova 2010 [10]

The first four columns describe juvenile rudiment development reported in the literature. The fifth column identifies which rudiments were reported to invaginate (and/or shown to invaginate in figures). The final column gives the total number of juvenile rudiments. cd—cephalic, discs td—trunk discs, dd—dorsal disc, cor—cerebral organ rudiments, pb—proboscis rudiment, cod—cerebral organ discs, dr—dorsal rudiment. ^a^ *Maculaura alaskensis* bears a typical planktotrophic pilidium and is included for comparison

Schmidt’s larva was originally described to form a juvenile via eight imaginal discs—paired cephalic, trunk and cerebral organ discs, and unpaired proboscis and dorsal rudiments—but Schmidt did not specify which invaginate from the epidermis [68]. Recent analysis with confocal microscopy shows only two pairs of imaginal discs, the cephalic and trunk discs, and a separate proboscis rudiment [17]. This technique also revealed a cluster of mesenchymal cells which, based on location, may contribute to the dorsal side of the juvenile, and similarly, another cluster of cells which appear to be associated with the formation of the cerebral organs [17]. More recently discovered non-feeding free-swimming pilidia have been studied in less detail. *Micrura rubramaculosa* and *M. verrilli* are thought to develop via five and *Micrura* sp. 803 via six imaginal discs, but development was only observed through their yolky epidermis, the discs were not identified, and disc formation was not described [15, 23]. So, while published literature suggests there are many possible departures from typical pilidial development in non-feeding larvae, this may be an artifact of the methods employed (e.g. histology vs. confocal microscopy), the depth of study, and interpretation by the author (e.g. which rudiments are counted as imaginal discs and which are not), rather than a representation of true developmental variation.

## Conclusions

In this study, the first to document in depth the development of a free-swimming non-feeding pilidium with modern microscopy methods, we have demonstrated that fundamental aspects of pilidial development (8 juvenile rudiments, catastrophic metamorphosis) are conserved in *pilidium nielseni*, the larva of a pilidiophoran species. Notably, its ciliary bands first form in segments along the transient lobes and lappets, resembling a planktotrophic pilidium, before connecting and encircling the larva as two transverse ciliary bands (Fig. 11) resembling a prototroch and telotroch of some spiralian trochophores. Patterns associated with the transition from planktotrophy to lecithotrophy predict its departures from typical pilidial development, including a larger egg size, an accelerated developmental timeline, a reduction in feeding structures (reduced lobes and lappets), and the rearrangement and repurposing of the ciliary bands (from feeding to locomotion). We suggest that transition from planktotrophy to lecithotrophy explains the trochophore-like morphology of *pilidium nielseni*, a compelling example of evolutionary convergence on a larval body plan often assumed to be widely homologous.

## Methods

### Collection of adults

We collected a total of 129 adults of *Micrura* sp. “dark” in rocky intertidal areas around Cape Arago in Charleston, Oregon (especially Middle Cove, 43.305 ˚N, 124.400 ˚ W) during or just prior to their fall/winter reproductive season. Of these, 33 were collected from October 2013 to March 2014, 41 were collected from July 2014 to February 2015, and 55 individuals were collected from July 2015 to March 2016. The increases in individuals collected from one spawning season to the next are likely due to our improved skill in locating them, rather than an increase in population. Fertile adults were observed from September through February. Some were fertile when collected, and others (particularly those collected in July and August) developed gametes in the laboratory following collection. Interestingly, despite being kept unfed in the laboratory for a year, a few males and one female developed gametes the next reproductive season. However, we were unable to start cultures with these males, and their sperm appeared somewhat lackadaisical. *Micrura sp.* “dark” were primarily found intertwined with the dense root masses of *Phyllospadix* spp. growing in shell hash, though several individuals were wedged between rocks, or in surf grass rooted in finer sand. Most individuals were collected from root masses of the most dominant surf grass, *Phyllospadix serrulatus,* but also from the root masses of *P. torreyi*, and possibly *P. scouleri. Micrura* sp. “dark” may be confused with two other common co-occurring and also undescribed lineiform nemertean species, which are similar in size (several centimeters long) and color (pinkish, reddish or brownish). Possible misidentifications include *Lineus* sp. “red,” which has considerably smaller oocytes (~100 μm) than *Micrura* sp. “dark” and develops via a planktotrophic pilidium [45], and Lineidae gen. sp. “large eggs,” a species with considerably larger oocytes (~600 μm) and encapsulated lecithotrophic development ([2], Maslakova, pers. obs.). *Micrura sp.* “dark” can be distinguished from these two species by the presence of a distinct caudal cirrus (a tail-like extension of the posterior end) (Fig. 2a), and by the nearly constant, pronounced peristaltic motion, which is especially apparent in the foregut region (Fig. 2b). One or two of these dramatic anterior to posterior peristaltic waves can be readily observed at nearly any given time, and their distinct margins conjure up images of a cartoon worm swallowing a series of doughnuts whole.

Initially, individuals were visually identified in the field prior to collection, and subsequently their identity was confirmed via DNA-barcoding (sequencing a 460–537 bp region of the 16S rDNA gene). Once we were confident and consistent in our identifications, confirming identification with DNA sequence data was no longer necessary. Adult individuals were photographed, and kept in 150 ml glass dishes in a flow-through sea table at ambient sea temperature, where their water was changed weekly.

### Obtaining gametes and rearing larvae

Gametes were dissected from gravid male and female *Micrura* sp. “dark” individuals when reproductive pairs were available, and we established a total of 18 cultures over three reproductive seasons from 2013–2016. In three instances, sperm was dissected from a male to fertilize naturally spawned oocytes, and in two others, naturally spawned oocytes and sperm were used. The 13 other cultures resulted from dissected oocytes and sperm. Observations are based on eleven embryonic cultures maintained through metamorphosis, including two started with spawned oocytes and one started with both spawned oocytes and sperm, as well as seven other cultures maintained through early developmental stages (two to three days), including one started with spawned oocytes, and another started with spawned oocytes and sperm. Oocytes were fertilized by a dilute suspension of sperm in filtered sea water (FSW, 0.2 μm), and cultures were maintained in 150 ml glass dishes of FSW placed in flowing sea tables at ambient seawater temperature. The water in their dishes was changed every one to two days. Because the first few cultures suffered a high mortality rate due to bacterial infestation, subsequent cultures were established and maintained in FSW re-filtered through a bottle-top vacuum system (Corning), and an antibiotic solution (a mixture of penicillin and streptomycin at a concentration of 5–50 μg/ml each) was added to the cultures.

### Light microscopy

Adult specimens of *Micrura* sp*.* “dark” were examined and photographed live using a Leica DF400 digital camera mounted to a Leica MZ10F dissecting microscope. Gametes and larval specimens were photographed, trapped between a glass slide and a coverslip supported by clay feet, using a Leica DF400 digital camera mounted to an Olympus BX51 compound microscope equipped with DIC.

### Fluorescent labeling and confocal microscopy

Larvae were relaxed in a 1:1 mixture of 0.34 M MgCl_2_ and FSW for 15 min, then in 100% 0.34 M MgCl_2_ for 15 min prior to fixation. They were fixed in 4% paraformaldehyde prepared from 16% or 20% ultrapure paraformaldehyde (Electron Microscopy Sciences) and filtered sea water. Fixed specimens were rinsed in three 10-min changes of phosphate buffered saline (PBS, pH 7.4, Fisher Scientific), then stored in PBS at 4 °C, or immediately permeabilized and stained. Larvae were permeabilized with three changes of PBS with 0.1% or 0.5% Triton X-100 (PBT) and rinsed in three 10-min changes of PBS. Specimens were stained with Bodipy FL phallacidin (Molecular Probes) at a concentration of 5 U/ml, propidium iodide (Sigma) at a 0.1% concentration, or a combination of both in 0.1% or 0.5% PBT. Stained specimens were rinsed in three 10-min changes of PBS, then stored in PBS at 4 °C, or immediately mounted. To view internal structures, specimens were mounted onto Poly-L-lysine (Sigma) coated coverslips, dehydrated through an isopropyl alcohol series (70%, 80%, 90%, 100% I, 100% II) for 40 s-1 min at each step, then cleared with three 10-min changes of Murray Clear (a 2:1 mixture of benzyl benzoate and benzyl alcohol). Slides were prepared with strips of foil tape to support the coverslip. After mounting, the coverslips were filled with Murray Clear, which has a refractive index close to that of the immersion oil (~1.5) used for imaging, then sealed with nail polish and imaged immediately, or stored at 4 °C. To view surface features, stained specimens were placed in a glass-bottom microwell dish filled with PBS, and covered with a coverslip.

Specimens were imaged with an Olympus Fluoview 1000 laser scanning confocal mounted on an Olympus IX81 inverted microscope. Specimens mounted in Murray Clear were imaged with a UPlanFLN 40× 1.3 NA oil lens. Uncleared specimens mounted in PBS were imaged using a UPlanFLN 40× 1.15 water lens. Stacks of 0.65 μm optical sections were imported into ImageJ 1.47v (Wayne Rasband, Nations Institute of Health, Bethesda, MD, USA) for further processing. Channels were false-colored and levels adjusted in Adobe Photoshop CS6. In figures, we refer to stacks of a subset of optical sections of a specimen (most often projections of three sections) as “slabs.”
